# Color Capable Sub-Pixel Resolving Optofluidic Microscope and Its Application to Blood Cell Imaging for Malaria Diagnosis

**DOI:** 10.1371/journal.pone.0026127

**Published:** 2011-10-11

**Authors:** Seung Ah Lee, Ricardo Leitao, Guoan Zheng, Samuel Yang, Ana Rodriguez, Changhuei Yang

**Affiliations:** 1 Department of Electrical Engineering, California Institute of Technology, Pasadena, California, United States of America; 2 Department of Microbiology, Division of Medical Parasitology, New York University School of Medicine, New York, New York, United States of America; 3 Department of Bioengineering, California Institute of Technology, Pasadena, California, United States of America; Texas A&M University, United States of America

## Abstract

Miniaturization of imaging systems can significantly benefit clinical diagnosis in challenging environments, where access to physicians and good equipment can be limited. Sub-pixel resolving optofluidic microscope (SROFM) offers high-resolution imaging in the form of an on-chip device, with the combination of microfluidics and inexpensive CMOS image sensors. In this work, we report on the implementation of color SROFM prototypes with a demonstrated optical resolution of 0.66 µm at their highest acuity. We applied the prototypes to perform color imaging of red blood cells (RBCs) infected with *Plasmodium falciparum*, a particularly harmful type of malaria parasites and one of the major causes of death in the developing world.

## Introduction

The optical microscope is an essential tool in biological science and medical diagnostics. The ability to interrogate the morphology of biological samples provided by microscopy enables the researchers and physicians to track dynamics, improve diagnosis and identify unknown organisms/cells. However, since its invention, optical microscopes have been constructed using lenses and optical components which make the systems bulky and expensive, rendering the method cumbersome for field diagnostics in rural areas[1], [2].

In recent years, there has been an increased interest in the miniaturization of microscopes and various methods have been reported for the construction of low-cost, hand-held imaging devices [3], [4], [5], [6], [7], [8]. The optofluidic microscope (OFM) is one such method [6], offering lensless imaging in the form of an on-chip device that is manufacturable through semiconductor foundries. The OFM achieves high resolution imaging by using microfluidic flow to scan specimens across submicron apertures on a complementary metal-oxide semiconductor (CMOS) imagesensor [6]. The scheme allows for high throughput imaging of microscopic specimens, particularly advantageous for imaging and screening a large quantity of samples dispersed in liquid. Recently, sub-pixel resolving OFM (SROFM) has been developed [7], with the incorporation of a pixel super-resolution algorithm [9], [10], which further reduces the device cost and improves the yield of imaged samples. This scheme removes the need for submicron apertures, instead taking and combining a sequence of low-resolution direct-projection images to reconstruct a resolution-enhanced image. SROFM has been reported to achieve resolution (at its highest acuity plane) comparable with that of a 20x objective microscope (0.40 NA, 0.85 µm resolution), suitable for imaging of biological specimens [7]. In addition to the high optical resolution, the use of microfluidic channels allows for the automated scanning of large numbers of samples in a fast and continuous manner similar to that of a flow cytometer.

The combination of high resolution imaging and flow-cytometry scheme implies that the OFM system may be suitable for diagnostic imaging of blood samples. For many diseases involving blood-borne parasites and blood cell deformations, the gold standard of diagnosis is with optical microscopy, where a large number of blood cells needs to be imaged with high optical resolution [11]. However, in rural and developing areas, microscopy-based diagnosis suffers from its bulkiness and cost, which hinder the eradication effort for poverty-related diseases like malaria [12]. For such challenging environments, portable “ipstick”devices based on immunochromatographic methods have been developed [13] with inherent limitations in the detection sensitivity and diagnosis accuracy [11]. SROFM combines the high sensitivity of microscopic diagnosis and the accessibility and low-cost of a portable device, offering a good potential alternative for the field diagnosis of malaria. Also, flow cytometry-like screening of blood sample can potentially provide a quantitative analysis of infection and classification of parasites. Previously, we have demonstrated monochromatic imaging of various biological samples using SROFM devices with a resolution of 0.75 µm at its plane of highest acuity [7]. In order to utilize the SROFM device in malaria diagnosis, color imaging with higher resolution would need to be achieved for detection and identification of the stained parasites within the RBCs.

Here we report on color imaging of blood samples with a SROFM device using color illumination. Our color SROFM has achieved a resolution of 0.66 µm. Here we used sequential red-green-blue (RGB) illumination to obtain a low-resolution sequence for each color and then combine them to a single high resolution color image. With this scheme we imaged both naïve and P. falciparum infected RBCs stained with Toluidine blue. We demonstrate a proof-of-concept on-chip device for imaging-based malaria detection.

## Results

### Design and Implementation

**Figure 1:**
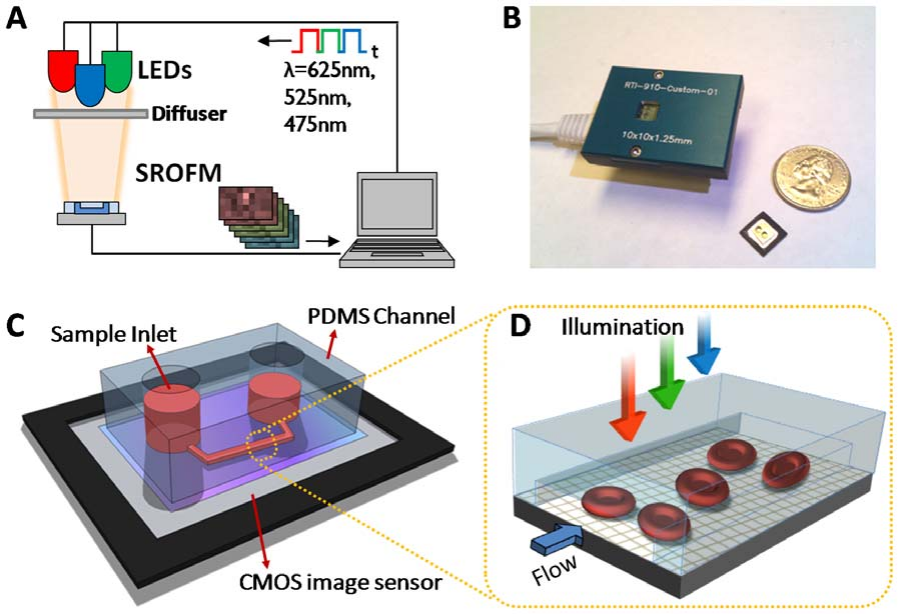
Schematics and prototypes of SROFM device. (a) Schematics of color SROFM system showing sequential RGB LED illumination and SROFM device controlled by a computer. (b) Current SROFM device composed of the CMOS sensor-based SROFM chip and camera head. The SROFM chip measures 1 cm 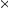 1 cm and the total device, 5 cm 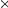 3 cm 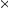 3 cm in size. (c) Schematics of a single SROFM chip composed of a PDMS microfluidic channel and a CMOS image sensor. (d) Detailed view of color imaging area where the sample, blood cells, is flowing in the channel on top of the sensor' pixel grid while the illumination is varied in color.

Our prototype color SROFM system is depicted in Fig. 1A. The illumination, image acquisition and image processing were controlled by a laptop computer. Three light emitting diodes (LED) with wavelengths of 625 nm, 525 nm and 475 nm were used for illumination with a switching rate of 8Hz. LEDs and the diffuser plate were placed about 5 cm above from the sensor in order to provide enough room for the pipetting the samples into the system. The illumination intensity of 0.3, 0.18 and 0.28 W/m^2^ was used for red, green, and blue, respectively. Fig. 1B shows the circuit board for the sensor (EPIX inc.) and a SROFM chip, which consisted of a CMOS image sensor and a poly-dimethylsiloxane (PDMS) microfluidic channel mounted on top (Fig. 1C), with a total size of approximately 1 cm by 1 cm. The pixel size of CMOS sensor used in all experiments was 2.2 µm. The chip is mounted on a commercial 5 megapixel camera with cat-5 Ethernet cable connection to a commercial PCI board (EPIX inc). For data acquisition, we used a commercial software (EPIX XCAP LTD) with 3GB frame buffer and the processing was performed with a custom software written in Matlab.

Imaging was performed in the following fashion. We flowed the sample through the microfluidic channel and used the sensor to take a sequence of direct projection images from underneath the channel. These direct projection images are intrinsically low in resolution - with the resolution limited to 4.4 µm or equal to 2 pixel widths. We next processed these images with image processing software to generate a high resolution image [7], [9]. The image processing software is comprised mainly of two parts – a cell tracing and a sub-pixel resolving algorithm. The movement of each blood cell in the raw sequence was traced in order to obtain the motion vector of each cell, a vector which describes the sub-pixel shift of each frame for rearrangement of each frame in the high resolution matrix. The sub-pixel resolving algorithm consists of shifting each low resolution frame by the relative sub-pixel shift given by the motion vector and adding all the frames all together to fill a high resolution image grid with the enhancement factor (EF) of n, where each n-by-n pixel area of the high resolution image grid was filled by n^2^ frames of a single 1-by-1 pixel area of the low resolution image grid. The reconstruction was performed only on the regions in low resolution sequence that contain blood cells.

The flowing of the sample through a microfluidic channel was a convenient mechanism for translating the specimen across the sensor to introduce sub-pixel shifts that are essential for the pixel super-resolution algorithm to function properly. Due to the laminar flow formed in the fluidic channel, the samples were carried in a translational movement with controlled speed. In order to scan the sample through two dimensions, we aligned the PDMS microfluidic channel with a small angle (7.5°) with respect to the pixel grid. We designed the microfluidic channel with a height of 5.5 µm so that the blood cells were translated near the surface of the active area of the sensor without rolling. In order to reduce in-plane rotation of the cells, inertial focusing of the blood cells was introduced by putting obstacles along the walls in the beginning part of the channel [14]. This reduced the number of cells near the channel walls which were more likely to rotate due to the asymmetric flow differentials near the channel walls. In our device, the PDMS microfluidic channel was pretreated with poly-ethylene glycol and heparin prior to use. This treatment facilitated the flow by enhancing the wettability and prevented cells from attaching to the surface, which can cause blockage of the channel and/or disturbance in the flow. In addition, an array of micropillars with diameter and spacing of 15μm were placed near the inlet to block any clumped cells from entering the channel. Detailed procedures of fabrication and pre-treatment are explained in the materials and methods section.

### Monochromatic Imaging of blood samples

**Figure 2:**
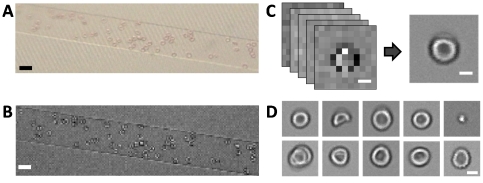
Monochromatic imaging of blood cells with SROFM. (a) Conventional bright field microscope image of the same field of view, taken with a 10x objective lens. (b) Low resolution image of blood cells in a SROFM device directly taken from the CMOS image sensor. (c) Low resolution sequence of a single RBC to a high resolution image converted with EF = 10. (d) Human blood cell images taken with SROFM. Scale bars indicate 20 µm in (a),(b) and 5 µm in (c),(d).

For imaging of blood cells, a blood sample was diluted to 1∶50 with Phosphate buffered saline (PBS), in order to prevent overlapping of the cells in the channel and heparin was added to a final concentration of 100 U/ml to prevent clogging. The pre-treated whole blood sample was injected into the inlet of the device and flowed through the channel. As the blood cells flowed across the channel, low resolution images were obtained from the image sensor with a region of interest (ROI) of 300 by 80 pixels. With our device design and pre-treatment conditions, a drop-and-flow scheme allowed for a flow rate of 500-1000 µm/s, with an average of approximately 50 cells within the imaging ROI at any time. The CMOS image sensor used in the experiment provided a maximum frame rate of 838 fps with the ROI of 300 by 80 pixels. We typically used 800fps with an exposure of 0.1ms to ensure that the motion-blur from the movement of the blood cells within a single frame was less than 100 nm. 100 low resolution frames were used for pixel super resolution reconstruction with an enhancement factor of 10. (Fig. 2c). With the current values of sample flow rate and low resolution imaging frame rate, the system can scan approximately 400 cells/sec for monochromatic imaging and 100 cells/sec for color imaging. This scan rate can be increased by further optimizing channel dimensions and sample concentrations. Fig. 2d shows typical images of blood cells acquired with our device.

**Figure 3:**
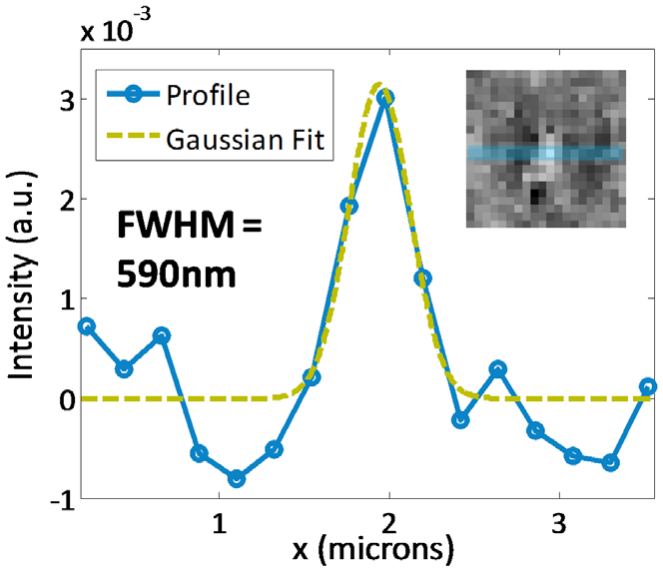
Optical resolution of our SROFM device. 500 nm microsphere imaged with 2.2 µm-pixel sensor (inset) shows that the device can resolve the lensing effect of the bead with a FWHM of 590 nm.

The optical resolution of the device was investigated by imaging 500 nm microspheres with our SROFM device. We used 500 nm blue-dyed microspheres (Polysciences) to enhance the contrast of the microspheres images. The microspheres were flown through a 1.5 µm-thick channel. As shown in Fig. 3, the bright centers of the microspheres were clearly resolved, with the full-width half maximum (FWHM) of 590 nm under EF of 10, suggesting the optical resolution of the device was 660 nm since two spots would need to be at least 3 high resolution pixels apart to be distinguished. Note that the FWHM value at EF = 10 has improved from our previous result of 800 nm to 590 nm, a result of the reduction of the sensor pixel size from 3.2 µm to 2.2 µm [7]. We further note that resolution deteriorates as the distance between the sample and sensor surface increases. Verification of this relationship has been examined in Ref. 6. By numerical analysis, we expect the point spread function with 500 nm microsphere will spread out to FWHM of 1.8 µm at the center of 5.5 µm channels. The resolution can be improved by using higher EF, which requires more precise control of sample movement over a longer scanning length. The reduction of sensor pixel size can also improve image resolution further. However, this improvement approach is expected to yield diminishing returns for pixel sizes beyond our current sensor pixel size, without a tandem effort to push the blood cells closer to the channel floor to mitigate the resolution deterioration associated with the sample-to-sensor-surface distance.

### Color imaging of malaria-infected red blood cells

In on-chip microscopy, color imaging can be a crucial component in order to detect and identify different functional structures within biological specimens with staining. Typically, color imaging can be done with the use of filters, by putting an array of RGB filters on the pixel grid of image sensors. However, since SROFM achieves highest resolution with samples flown near the sensor's surface, we removed the additional filter layer between the sample and the sensor and, instead, accomplished color imaging by using three-color sequential illumination. In our color SROFM scheme, RGB LEDs were used with sequential switching to generate separate color image sequences, which were later combined into a single full-color high resolution image.

In the experiment, we alternated the RGB light sources at a switching rate of 8 Hz. The sensor acquired images at a rate of 800 fps. This allowed us to acquire 100 low-resolution frames between each color switch. We then processed each 100 frame set to generate a high resolution image with the enhancement factor of 10. The center-of-mass of each cell was tracked by motion vector calculation and we used that as a reference point for image registration. To constitute a color image, we simply superposed the R, G, and B images by overlapping their center-of-mass. To check for mis-registration, we also superposed 2 sequential R images by the same approach and check for image shift/distortion.

**Figure 4:**
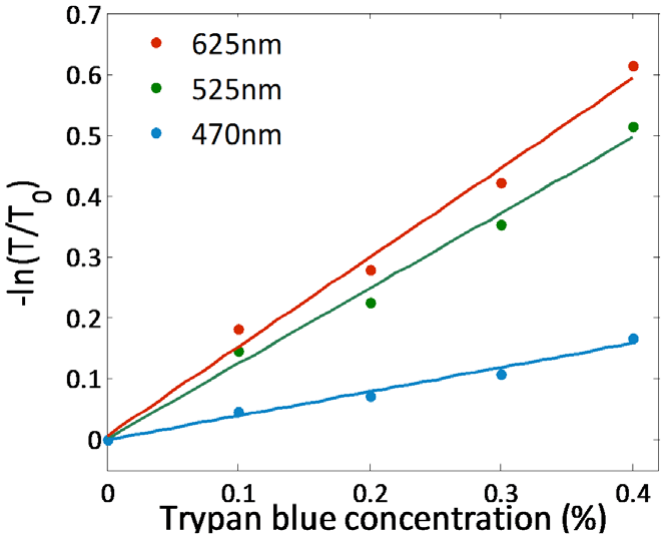
Color response of our SROFM device. Light transmission through a 27 µm channel containing different concentrations of Trypan blue dye. This figure demonstrates agreement with the Beer-Lambert law of light absorption, validating SROFM's color imaging capabilities.

To verify the color capability of the RGB illumination method with SROFM, we measured the light transmission through the microfluidic channel with different concentrations of Trypan blue dye. By the Beer-Lambert law, light transmission through an absorbing dye can be expressed as:
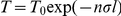
where *T_0_* is the light transmission in the absence of a sample, *n* is the concentration of the dye, σ is the absorption cross-section of the dye and *l* is the optical path length through the sample. Signal through the 27 µm-channel filled with the Trypan blue dye with concentration varying from 0 to 0.4% was measured for all three illumination wavelengths of 625 nm, 525 nm and 475 nm. With increasing concentration of dye, we expect 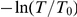 o increases linearly too, with the slope corresponding to the absorption cross-section σ for each illumination wavelength. Fig. 4 confirms that the logarithm of signal transmission through Trypan blue dye is proportional to the concentration of the dye and that the relative values of σ for the three wavelengths agree with the known absorption spectrum of Trypan blue dye.

To demonstrate the potential application of our color SROFM device for malaria field diagnostics, we imaged red blood cells (RBCs) infected with *P. falciparum*. Imaging-based diagnosis of Giemsa-stained blood is the most widely used diagnosis method for malaria in the field [11]. However, to avoid additional processing steps in sample preparation, we searched for a dye that, unlike Giemsa, does not require previous fixation of cells. We tested Toluidine blue at different concentrations to find a protocol compatible with SROFM, finding that a 2% Toluidine blue solution provides adequate staining of intracellular *P. falciparum* with low background in the red blood cell cytosol without the need for washing the cells. In order for SROFM to be utilized for the diagnosis of malaria in the field, the ability to detect the color-stained parasites in high resolution is necessary since identification of plasmodium species by appearance is an essential part of the diagnosis for the treatment and the control of the disease.

**Figure 5:**
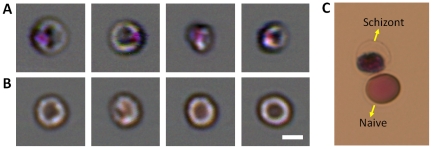
Color SROFM images of *P. falciparum* -infected and naïve RBC. (a) Color images of *P. falciparum* schizont-stage RBCs stained with Toluidine blue, showing a distinct purple spot within each RBC, which appears as dark spots in the green channel. (b) Color images of naïve RBCs show clear differences with the infected RBCs. Naïve RBCs were also stained with Toluidine blue as a control. (c) 100x Bright field microscope images of *P. falciparum* schizont-stage and Naïve RBCs stained with Toluidine blue. All scale bars indicate 5 µm.

In this experiment, we purified cultures of *P. falciparum* to obtain infected RBCs (iRBCs) in the schizont or late trophzoite stage at a concentration >98%. Samples were treated with 2% Toluidine blue, to stain the *P. falciparum* parasites within. The samples were live-stained in solution, as opposed to using blood smears. Cells were imaged without washing of the dye, since the background color contribution from the staining solution is negligible due to the small thickness of the channel. Fig. 5 shows color images of *P. falciparum* iRBCs at the schizont-stage and naïve RBCs taken with the SROFM device. The images show clear differences between the schizont-stage and naïve RBCs, with the bright purple spots in the iRBCs indicating the presence of parasites. Additionally, we conducted the same experiment with *P. falciparum* ring-stage iRBCs, but we were not able to achieve a clear differentiation in that experiment. We believe that the unsuccessful identification of *P. falciparum* ring-stage iRBCs possibly resulted from the weak color signal from the live-stained blood samples.

As an initial study for the diagnostic potential of this technology, we performed a blind experiment with SROFM images of naïve and schizont-stage RBCs. In the experiment, we asked 4 experienced malaria researchers to identify the cell type for 30 randomized images of naïve and schizont stage erythrocytes (13 and 17 images). The researchers were able to make the right identification in 88% of the cases. The current result of successfully detecting schizont-stage iRBCs is not yet sufficient for diagnosing *P. falciparum* infections, which require detecting the earlier ring-stage iRBCs. However, the results suggest that the SROFM may be able to identify other types of plasmodium parasites that exist in the schizont- stage in peripheral blood of patients. *P. vivax* is an excellent example as this variety can be diagnosed from schizont-stage iRBCs.

## Discussion

We have developed a compact high-resolution color imaging microscopy device based on sub-pixel resolving optofluidic microscopy (SROFM) and color illumination. Our device achieved an optical resolution of 660 nm. Our preliminary experiment indicates that color SROFM can potentially be a useful diagnostic tool for identifying malaria infected cells.

One advantage of the SROFM technique for malaria diagnosis is that it can operate with a simpler preparation protocol than standard microscopy based on blood smears. A typical blood smear test requires several steps in film preparation, fixation, staining and washing. In contrast, the SROFM technique employs solution-based preparation protocol where the imaging is directly performed on the samples without requiring slide preparation. The sample preparation protocol for SROFM imaging comprises of a single step of mixing a blood sample with a solution containing buffer, anticoagulants and stain. While a typical blood smear requires a washing step to remove excess dye that was not uptaken by the sample, we found that such a step is not required in our imaging process with the SROFM. This is because the overall height of the microfluidic channel was sufficiently low that the residual dye in the fluid medium did not significantly attenuate light transmission when compared to the light attenuation from the dye within the cells.

We believe that SROFM technique can be a good fit with the technological needs of global healthcare. Portable imaging/diagnostic devices can benefit for healthcare in rural regions where conventional clinical devices are too bulky to transport well or too expensive to employ widely. In addition, electronic devices that can be integrated with telemedicine network can support efficient utilization of scarce medical expertise. For this matter, we believe that the SROFM technique possesses a few key potential advantages in global health applications; (1) Compact size of the SROFM technology allows for the implementation of light-weight and cheap hand-held systems. (2) The technique requires minimal training for operation and the collected raw data can be processed into useful microscopy images with none or very few manual inputs. (3) The principle of operation is inherently simple and robust and thus has a relatively low demand on the computational power it requires for image processing. Although the data acquisition of current SROFM system relies on an Ethernet cable connection and a frame buffer memory in the processor, a new camera system with an on-board memory and a universal serial bus interface can support stable acquisition and transfer of data with low-power portable systems. (4) The final image data is obtained for each individual cells with consistent image parameters, a convenient format for quantitative analysis and application in computer aided diagnosis based on pattern recognition. As such, this technology is potentially an attractive candidate for telemedicine systems in the developing world.

However, further improvement of the technology would need to occur for the method to be truly applicable to malaria diagnostics. For example, SROFM would need to improve in its sensitivity to the point where *P. falciparum* ring-stage iRBCs can be observed. Smaller pixel size may increase the imaging performance of SROFM device. Alternately, a better dye protocol by which stronger staining of iRBCs would need to be developed. In addition to improving its performance, thorough assessment is needed to determine whether conventional criteria for malaria diagnosis applies for SROFM images, since they may differ from those obtained with conventional light microscopy. Total cost per diagnosis need to be considered in order to compete with current techniques; there is room for cost reduction with the lowering price of CMOS sensors, integration of multiple tests on a single chip and foundry-based mass-production of fluidic channels. However, other practical issues such as reagent shelf-life and the sensor's durability and in extreme environments also need to be thoroughly assessed. Along with the technological improvements discussed above, a field-testing of the system will provide better perspective on the aptitude of the technique in the global malaria diagnostics market.

## Materials and Methods

### Fabrication of SROFM devices

A 2.2 µm pixel CMOS sensor (Aptina MT9P031I12STC) was used in all experiments. The color filter array and microlens array inherent in the sensor were removed via oxygen plasma cleaning at 150 W for 10 min. Poly-dimethyle siloxane (PDMS) microfluidic substrate was fabricated by conventional soft lithography as described in ref [7]. The PDMS channel was 50 µm in width and 5.5 µm in height, and was chemically treated with Poly-ethylene glycol(PEG) and UV exposure to render the surface hydrophillic, with the recipe decribed in ref [15]. In addition, the surfaces were heparinized by flushing the channel with 100 units of heparin in PBS.

### Preparation of samples

*P. falciparum* 3D7 strain was cultured *in vitro* following standard procedures. Mature *P. falciparum*-infected erythrocytes, including schizonts and late throphozoites, were isolated from the interphase of a 90%/40% Percoll gradient. As control, uninfected erythrocytes were prepared in parallel. After isolation, cells were diluted to ∼ 50000 cells/ml in PBS with 100 units of heparin. This dilution prevents overlapping of the cells in the channel. Then the sample was incubated with 2% Toluidine blue for 10 min before imaging. Human whole blood sample used in all experiments were obtained from New York Blood Center.
